# Spontaneous Ureteral Rupture Diagnosis and Treatment

**DOI:** 10.1155/2013/851859

**Published:** 2013-12-23

**Authors:** E. Pampana, S. Altobelli, M. Morini, A. Ricci, S. D'Onofrio, G. Simonetti

**Affiliations:** General Hospital Tor Vergata, PTV Foundation, Diagnostic Imaging, Molecular Imaging, Radiotherapy and Interventional Radiology Department, Oxford Street 81, 00133 Rome, Italy

## Abstract

Rupture of the urinary collecting system associated with perinephric or retroperitoneal extravasation of the urine is an unusual condition and it is commonly associated with renal obstructing disease. Perforation could occur at any level from the calix to the bladder but it is usually seen at the fornices and upper ureter. It may lead to several serious consequences including urinoma, abscess formation, urosepsis, infection, and subsequent irreversible renal impairment. We report a case of a 69-year-old woman who presented at the emergency department of our institution with severe abdominal pain. Due to symptomatology worsening, complete laboratory evaluation was performed and the patient underwent abdominal contrast enhanced computed tomography (CT) evaluation which showed contrast agent extravasation outside the excretory system without any evidence of renal calculi at basal acquisition. It was decided to perform a double-J stent placement which was followed by complete healing of the ureter and its removal was performed 8 weeks later. Diagnosis and therapeutic approaches are discussed.

## 1. Introduction

Obstruction of the genitourinary system is followed by an increase of intraluminal pressure which may lead to rupture of the collecting system and consequently urine extravasation with urinoma formation. Spontaneous rupture of the ureter is rare and can be traumatic or nontraumatic. Calculi represent the most frequent cause of ureteral and pelvis rupture in the nontraumatic group. Urine extravasation may be clinically occult or may lead to symptoms of acute abdomen. We report a rare case of spontaneous ureteric rupture in a patient with a clinical history of recurrent renal colics in the younger age treated with double-J stent placement. We further describe this minimally invasive interventional approach.

## 2. Case Presentation

A 69-year-old woman was admitted to the emergency department of our institution with a severe right-sided flank pain that started 6 hours before, nausea, and history of renal calculi in the younger age. In suspicion of an episode of renal colic, analgesic drugs therapy and complete laboratory evaluation were performed. Her vital signs were as follows: heart ratio: 89 beats per minute and regular; blood pressure: 145/90 mmHg; respiration: 18 per minute; and body temperature: 36,8°C. She had leukocytosis in the blood (9,300/mm^3^), with 48,2% of neutrophils. Urine analysis showed the presence of leukocytes and erythrocytes. Other values were within the normal limits.

The patient reported a previous episode of colic pain 6 weeks before which was spontaneously resolved. Symptomatology worsened in time; thus, abdominal computed tomography was mandatory.

**Figure 1:**
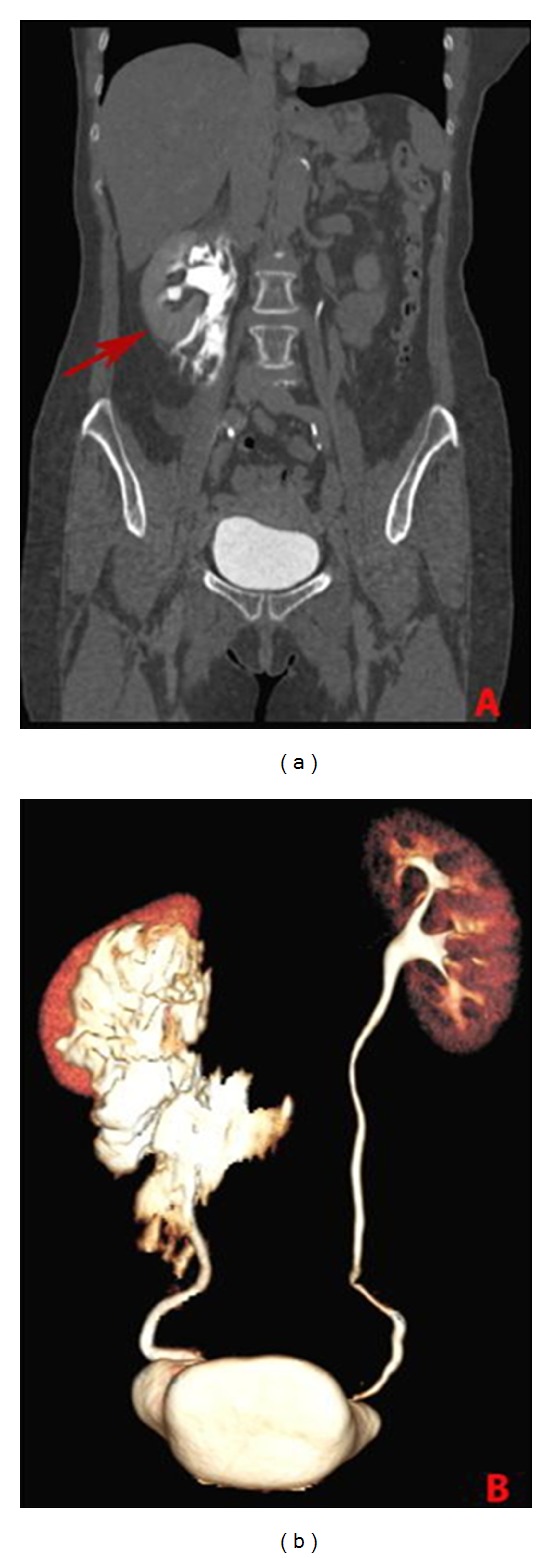
Contrast enhanced CT coronal image (a) and VR reconstruction (b) in the delayed phase documenting contrast extravasation extension (arrow).

Abdominal CT with endovenous administration of contrast medium (Iopromid 370, Ultravist, Schering, Germany) was intended to get a complete overview of the entire excretory system and showed in the delayed phase (10 minutes after contrast medium injection) a late filling of the right lower ureter and a perinephric fluid collection. Contrast medium extravasation extended from the vertebral body of L2 to L5 and crossed the midline at the level of the aortic bifurcation (Figure 1).

No evidence of factors that caused ureteric rupture was identified at CT evaluation.

**Figure 2:**
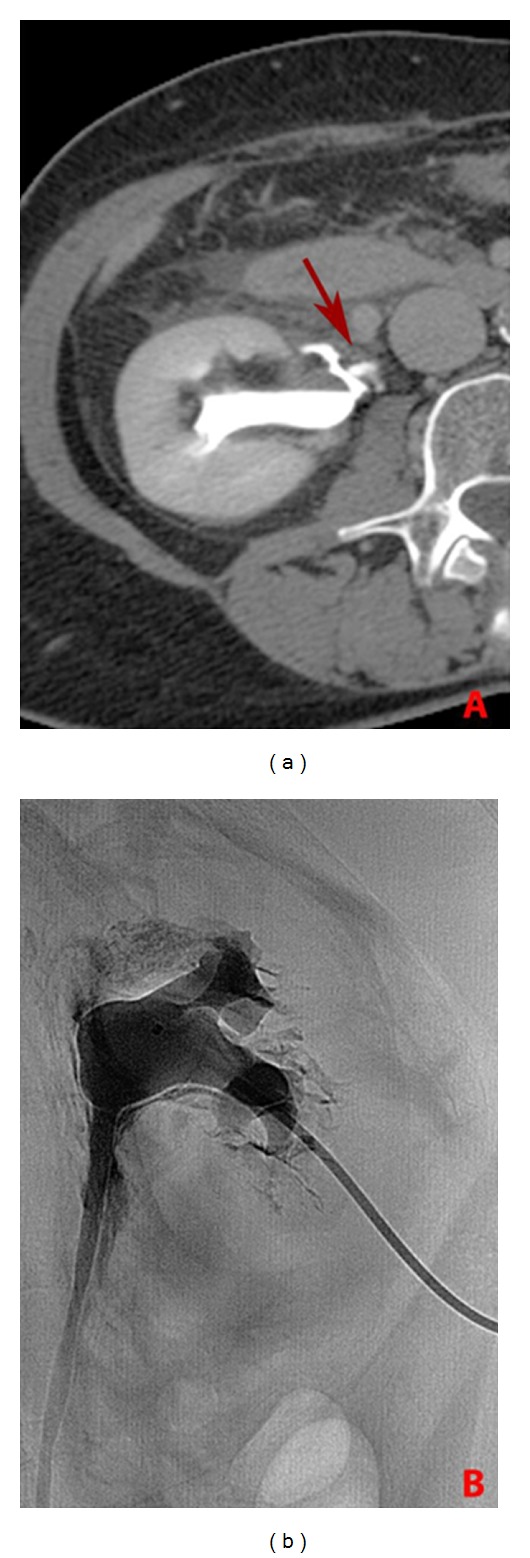
Axial contrast enhanced CT image (a) and fluoroscopic view (b) showing the site of rupture of the excretory system (arrow).

**Figure 3:**
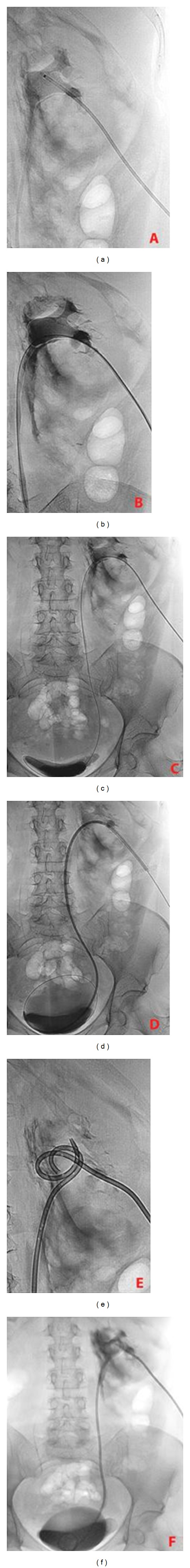
(a) Preprocedural urographic control; (b, c) advance of a stiff guidewire through the ureter in the bladder; (d) double-J stent placement; (e) diversionary protection nephrostomy positioning; and (f) postprocedural urographic control.

Two hours later the lower calyceal group of the right kidney was punctured with Chiba needle (Neff Percutaneous Access Set, Cook Medical Inc., Bloomington, U.S.A.) under ultrasonographic guidance. Contrast media injection under fluoroscopy confirmed the presence of an extravasation localized in proximity of the pyeloureteral junction (Figure 2). After the placement of a stiff guidewire through the ureter in the bladder, an 8 French double-J stent (Flexima Regular 8 Fr, Boston Scientific) was positioned and postprocedural control showed correct placement of both its proximal and distal extremities. At the end of the procedure a diversionary nephrostomy catheter of 8 French was placed in renal pelvis (Figure 3). There was no evidence of intra- and postoperative complications. Double-J stent was removed 8 weeks later after complete healing at the site of rupture.

## 3. Discussion

Urine extravasation is the result of a leakage of the urinary collecting system at any level from the calix to the urethra. This is defined as “spontaneous” if it is not induced by external trauma, iatrogenic manipulation, degenerative kidney diseases, or previous surgery.

First cases were described in 1856 by Diaz and Buenrostro [1]. Ruptures of the ureter are rare and can be traumatic or nontraumatic. Calculi represent the most frequent cause of ureteral and pelvis rupture in the nontraumatic group. Other causes of collecting system obstruction that may lead to rupture are congenital abnormalities, abdominal or pelvic masses, retroperitoneal fibrosis, iatrogenic or postirradiation strictures, transplanted kidney, and connective tissue disorders [2–4].

Lymphoma and chemotherapy have been also described as a rare case of renal pelvis rupture. Usually collecting system rupture is monolateral, but Niggeman et al. [5] described a case of bilateral spontaneous fornices rupture.

Pathogenetic pathway underlying urinary collecting system nontraumatic disruption can be identified in an increase of intraluminal pressure. The fornix is the most common site of rupture followed by the upper ureter when pressure exceeds a critical level reported from 20 to 75 mmHg. Urine extravasation may lead to urinoma formation [6, 7]. This could be confined, could be encapsulated, or may manifest as free fluid, as in our case. Most urinomas involve the perirenal space within the Gerota fascia, but if extensive they can cross the midline.

Symptomatology usually could not be differentiated from a renal colic but sometimes could mime an acute abdomen. In some cases it could be very difficult due to lack of symptoms.

Differential diagnosis includes diverticulitis, cholecystitis, appendicitis, and others.

Ultrasonography represents the first line of investigation for renal colic [8]. It can identify the presence of hydronephrosis, calculi within the renal pelvis, and perinephric urinoma, which appears as well-defined clear fluid collections.

In our case, ultrasonography was not performed because we preferred to perform immediately abdominal contrast enhanced CT due to symptomatology worsening and to gain a panoramic view of the entire excretory system. Computed tomography is the technique of choice in the diagnosis of urinary collecting system leaks and urinomas. The latter could appear as confined, encapsulated fluid collections or as free fluid. Delayed acquisitions (5–20 minutes after contrast medium administration) are mandatory to identify the attenuation increase of the urinoma which can range from 0 to 20 HU before intravenous contrast administration and then enhance up to 200 HU after contrast administration [9, 10]. Reformatted coronal, sagittal, and volume rendering images well depict the extension of the collection which as in our case can reach and cross the midline (Figure 1(b)) [11].

Treatment options included surgery or interventional radiology [12–14] and should be individualized in each case.

In this case, a minimally invasive procedure can consist in percutaneous urinoma drainage using a nephrostomy catheter, with a double-J stent placement. Fluoroscopy permits to achieve an unobstructed urinary outflow, the healing of the perforation, and the regression of the urinoma in the majority of cases. Its placement may be performed either retrograde through the bladder or antegrade, as in our case, through a percutaneous nephrostomy [15]. To facilitate stent deployment, a mixed approach (anterograde and retrograde) could be used in collaboration with the urologist [16].

A diversionary nephrostomy catheter could be left in place to gain an access to the collecting system so that contrast medium could be injected to confirm the ureteral injury has completely healed.

As the incidence of late complications after surgery such as ureteric stricture, ureteropelvic stenosis, or periureteric fibrosis remains unknown, percutaneous interventional minimally invasive techniques are the first choice of treatment option nowadays. Spontaneous rupture of the ureter should always be considered in the differential diagnosis of a patient presenting with complex symptoms after renal colic.
